# Assessment of Bacterial Communities in Raw Milk Cheeses from Central Poland Using Culture-Based Methods and 16S rRNA Amplicon Sequencing

**DOI:** 10.3390/foods14244288

**Published:** 2025-12-12

**Authors:** Nikola Maciejewska, Anna Szosland-Fałtyn, Beata Bartodziejska

**Affiliations:** Department of Food Quality in Lodz, Prof. Waclaw Dabrowski Institute of Agricultural and Food Biotechnology—State Research Institute, 36 Rakowiecka St., 02-532 Warsaw, Poland; anna.szosland@ibprs.pl (A.S.-F.); beata.bartodziejska@ibprs.pl (B.B.)

**Keywords:** artisanal cheese, DNA 16S rRNA amplicon sequencing, lactic acid bacteria (LAB), microbial diversity, microbiological safety, raw milk, “twarog” cheese

## Abstract

Traditional raw milk cheeses are valued for their distinctive sensory properties and microbial richness but may pose microbiological safety risks. The objective of this research was to investigate the bacterial communities present in cheeses sourced from dairies from different regions of one voivodeship using culture-based methods and 16S rRNA amplicon sequencing. Artisanal Polish “twarog”-type cheeses produced from raw cow’s milk were obtained from four small local dairies in the Łódź Voivodeship. Microbial diversity and safety were assessed by culture-based enumeration following ISO standards and 16S rRNA amplicon sequencing targeting the V3–V4 region. Microbial counts included total viable microorganisms, lactic acid bacteria (LAB), *Enterobacteriaceae*, coagulase-positive staphylococci, yeasts, molds, and pathogens (*Salmonella* spp., *Listeria monocytogenes*). Significant differences (*p* < 0.05) were observed among cheeses, with samples ZJ-473 and ZJ-505 showing the highest LAB and total counts, while *Salmonella* spp. and *L. monocytogenes* were undetected. 16S rRNA amplicon sequencing revealed dominance of *Firmicutes* and *Proteobacteria*, mainly *Lactococcus lactis*, *Leuconostoc pseudomesenteroides*, and *Lactobacillus delbrueckii*. Alpha diversity and co-occurrence analyses indicated higher microbial complexity in samples ZJ-473 and ZJ-505. The integration of culture-based and sequencing data provided a comprehensive view of cheese microbiota and safety, confirming the protective role of LAB and emphasizing the need for strict hygiene in artisanal cheese production.

## 1. Introduction

Fermented dairy products have long been part of the human diet, with archeological evidence of fermentation practices dating back to 7000 BC in ancient China. In recent years, their popularity has surged due to growing awareness of their nutritional and health benefits, particularly their positive effects on gut microbiota and potential to improve life expectancy. Fermentation processes not only enhance the bioavailability of nutrients but also reduce harmful compounds such as lactose and galactose, making these foods suitable for individuals with lactose intolerance [1,2]. Parallel to this trend, consumer interest in organic foods has increased significantly, driven by concerns over health, environmental sustainability, and the desire to avoid synthetic additives and genetically modified organisms [3]. Moreover, traditional and regional food products have gained increasing popularity among consumers in Poland over the past several years. These products are frequently associated with health benefits and distinctive sensory qualities. The growing interest in natural and authentic flavors has encouraged many producers to develop artisanal products from raw milk [4]. One such product is “twarog”, a fresh acid-set cheese obtained through the natural acidification of milk by indigenous lactic acid bacteria (LAB) [5]. The consumption of raw milk cheese remains a subject of debate due to the potential presence of foodborne pathogens; however, cheeses made from pasteurized milk may also carry microbiological risks, often at levels comparable to, or slightly lower than, those associated with unpasteurized milk [6,7,8]. The microbiological safety of cheese largely depends on the microbial quality of the raw milk and the conditions maintained throughout the various stages of production. Ensuring high hygiene standards is essential in dairy processing. Such practices are detailed, among other sources, in the European Guide to Good Hygiene Practices for the production of artisanal cheeses and dairy products [9].

Raw milk cheeses exhibit a distinct advantage over their pasteurized counterparts, characterized by a more pronounced and complex flavor profile. This enhanced sensory quality is primarily attributed to the native microbiota naturally present in unpasteurized milk. Pasteurization negatively affects cheese quality by deactivating key enzymes and eliminating the native microbiota essential for flavor and texture development. Raw milk cheeses, in contrast, contain more volatile compounds like acids, esters, and alcohols due to microbial fermentation, and their sensory traits vary with microbial diversity, processing methods, and seasonal factors, resulting in greater flavor and texture complexity than pasteurized cheeses [8,10,11,12].

Certain LAB naturally occurring in raw milk produce bacteriocins, antimicrobial peptides synthesized by the ribosome, which suppress the growth of pathogenic microbes. Based on their structural and functional properties, bacteriocins are categorized into three main classes. Bacteriocins such as nisin, produced by *Lactococcus lactis*, have been extensively studied for their activity against foodborne pathogens like *Staphylococcus aureus* and *Listeria monocytogenes*. Likewise, *Enterococcus* species isolated from dairy environments can produce bacteriocins with strong anti-*Listeria* effects [8,13]. In addition to bacteriocins, LAB synthesize a range of antimicrobial compounds, including organic acids and hydrogen peroxide, which contribute to the inhibition of pathogenic microorganisms. *Lactococcus garvieae*, frequently isolated from raw milk cheeses, contributes to microbial stability by producing bacteriocins and hydrogen peroxide, which inhibit pathogens such as *S. aureus*. Despite its association with infections in animals and humans, its presence in cheese has shown beneficial effects without compromising sensory quality. *Enterococcus* spp., notably *E. durans*, play a functional role during cheese ripening by producing antimicrobial compounds effective against *Bacillus cereus*, *S. aureus*, *Escherichia coli*, *L. monocytogenes*, and *Enterococcus faecalis*, while also enhancing flavor development. *Hafnia* spp., such as *H. alvei*, are involved in flavor formation through sulfur compound production and act as adjunct ripening cultures. They support microbial balance by suppressing *E. faecalis* and *E. coli* O26:H11, highlighting their potential as functional cultures in raw milk cheese production [8,13,14,15,16]. Recent studies have provided updated insights into the microbial diversity, ecological interactions and safety-related characteristics of traditional raw-milk cheeses, broadening current understanding of how cheese-associated microbiota develop and influence product quality and safety [17,18,19,20,21].

To the best of our knowledge, this study represents the first application of metataxonomic analysis to examine the microbial diversity of raw milk cheeses produced in Poland across various farms. Therefore, the objective of this research was to investigate the bacterial communities present in cheeses sourced from dairies from different regions of one voivodeship using culture based method and 16S rRNA Amplicon Sequencing. In this study, we focused on artisanal raw-milk “twarog”-type cheeses produced in the Łódź Voivodeship, which we considered a representative area of Central Poland. The Łódź region is located in the geographical center of the country and belongs to the central Polish macroregion in administrative and agricultural statistics. It is characterized by a temperate climate, mixed crop–livestock farming, and a high share of small family-operated dairy farms, which together reflect the typical production conditions of Central Poland. Moreover, traditional fresh “twarog” made from raw cow’s milk is still widely produced and consumed in this voivodeship, using natural acidification driven by indigenous lactic acid bacteria rather than commercial starter cultures. These features make the Łódź Voivodeship a suitable model region for characterizing microbial diversity and microbiological safety of artisanal raw-milk cheeses produced in Central Poland.

## 2. Materials and Methods

### 2.1. Materials

Raw milk cheeses—“twarogs”—were purchased directly from the 4 local farmers in the Łódź Voivodeship in Poland. The Łódź Voivodeship was selected because it is centrally located and representative of the wider Central Poland region in terms of agricultural landscape and traditional dairy practices. The region has a long-standing tradition of producing raw-milk “twaróg”, and its environmental and production conditions reflect those typical of central Polish artisanal dairies. Restricting sampling to one voivodeship ensured ecological comparability among sites, enabling us to characterize within-region variability without introducing confounding inter-regional differences. Cheese samples were purchased using a randomized consumer-like sampling strategy. The acquisitions were carried out on different days at each dairy in the same manner as regular customers obtain these products, ensuring that the samples represented typical cheeses available for sale. The participating dairies were small family-operated farms with herd sizes ranging from 8 to 25 cows, corresponding to an estimated daily milk production of approximately 60–180 L, which is characteristic for such small-scale operations. Raw milk used in cheese production underwent routine on-farm quality control. Both somatic cell count (SCC) and total bacterial count (TBC) were regularly monitored by the producers, and the results were consulted with our research unit to verify compliance with hygiene requirements before sampling. Only milk meeting these criteria was used for the cheeses analyzed in this study.

The four dairies included in this study were selected to reflect the typical structure of artisanal “twaróg” production in the Łódź Voivodeship. Artisanal raw-milk cheeses in this region are produced almost exclusively by small, independently operating family farms, and there is no centralized or standardized system of production that would homogenize microbial profiles across dairies. The participating farms were geographically dispersed within the voivodeship and operated under distinct yet representative on-farm conditions, allowing the sampling design to capture the natural variability characteristic of regional artisanal cheesemaking. For this reason, cheeses obtained from these four dairies provide an ecologically meaningful representation of the microbial diversity found in artisanal raw-milk “twaróg” produced in the Łódź Voivodeship.

Cheeses were manufactured employing traditional cheesemaking protocols. Raw cow’s milk from small-scale farms was filtered through cheesecloth and kept at ambient temperature (20–25 °C) until natural coagulation occurred, usually within 24–26 h. The curd was then cut into small pieces to enhance whey expulsion and transferred to cheesecloth for gravity drainage at 12–15 °C over 12–14 h. After production, cheeses were transported under refrigerated conditions to the laboratory, where they were analyzed within 2 h of delivery.

### 2.2. Culture-Based Methods

Analyses of cheese samples were conducted to assess the presence of *Salmonella* spp., *L. monocytogenes*, total count of microorganisms (TCM), *Enterobacteriaceae*, coagulase-positive staphylococci, LAB, yeasts, and molds, in accordance with ISO standard methodologies. “Twarog” cheese samples underwent microbiological examination promptly after transportation to ensure accurate assessment of microbial status. Samples weighing 25 g were placed in filter bags, supplemented with 225 mL of Half-Fraser Broth (Oxoid, Basingstoke, UK) for the detection of *L. monocytogenes* or Buffered Peptone Water (BPW) (Oxoid, Basingstoke, UK) for other analyses, and homogenized for 60 s using a stomacher. Decimal dilutions were prepared using Peptone Water (PW) (Oxoid, Basingstoke, UK), and 1 mL of the appropriate dilutions was plated onto selective media. The enumeration of TCM was carried out using Plate Count Agar (PCA) (Oxoid, Basingstoke, UK). The inoculated plates were incubated at 30 ± 1 °C for 72 h, in accordance with ISO 4833-1 [22]. Mesophilic LAB were quantified by incubating de Man, Rogosa, and Sharpe (MRS) (Oxoid, Basingstoke, UK) agar plates at 30 °C for 72 h, following the ISO 15214 protocol [23]. Yeasts and molds were cultivated on chloramphenicol agar (Ch) (Oxoid, Basingstoke, UK) and incubated at 25 ± 1 °C for five days, as described in ISO 7954 [24]. *Enterobacteriaceae* were enumerated on Violet Red Bile Glucose Agar (VRBGA) (Oxoid, Basingstoke, UK), with incubation at 37 ± 1 °C for 24 h, according to ISO 21528-2 [25]. Coagulase-positive staphylococci were estimated by streaking samples onto Rabbit Plasma Fibrinogen (RPF) agar with supplement (Oxoid, Basingstoke, UK), followed by incubation at 37 ± 1 °C for 48 h, based on ISO 6888-2 [26]. The detection of *Salmonella* spp. was conducted in compliance with ISO 6579-1 standard [27], this involved a pre-enrichment step in Buffered Peptone Water (BPW), followed by selective enrichment in Rappaport Vassiliadis *Salmonella* (RVS) broth (Oxoid, Basingstoke, UK) and Müller–Kauffmann Tetrathionate–Novobiocin (MKTTn) broth (Oxoid, Basingstoke, UK). Subsequent streaking from both enrichment broths was performed onto Xylose Lysine Deoxycholate (XLD) (Oxoid, Basingstoke, UK) and Hektoen Enteric Agar (Oxoid, Basingstoke, UK), with incubation at 37 ± 1 °C for 24 h. The presence of *L. monocytogenes* was determined in accordance with ISO 11290-1 [28]. Cheese samples were first homogenized in Half-Fraser pre-enrichment broth and incubated at 30 ± 1 °C for 24 and 48 h. This was followed by selective enrichment in Fraser broth (Oxoid, Basingstoke, UK) at 37 ± 1 °C for an additional 24 h. Cultures were then plated onto Agar Listeria according to Ottaviani and Agosti (ALOA) (Oxoid, Basingstoke, UK) and OXFORD agars (Oxoid, Basingstoke, UK), incubated at 37 ± 1 °C for 48 h, with preliminary growth evaluation after 24 h.

### 2.3. DNA Isolation

For DNA isolation, the MagnifiQ™ 1 Genomic DNA Instant Kit (A&A Biotechnology, Gdynia, Poland; Product Code: 604A-1V-32) was used. This kit is based on magnetic bead technology and enables rapid and efficient purification of high-quality genomic DNA from various sample types. The procedure was carried out according to the manufacturer’s instructions using an automated extraction system, minimizing manual errors and the risk of contamination. The isolated DNA was of sufficient purity and quality for downstream molecular analyses. DNA quality was assessed quantitatively and qualitatively prior to library preparation. DNA concentration was measured fluorometrically using the Quant-iT™ PicoGreen™ dsDNA assay (Thermo Fisher Scientific, Waltham, MA, USA). The obtained concentrations were as follows: 7.27 ng/µL (ZJ-473), 3.62 ng/µL (ZJ-504), 12.37 ng/µL (ZJ-505), and 12.51 ng/µL (ZJ-513). All isolates fulfilled the minimal input thresholds required for 16S metagenomic library construction and passed the initial NGS quality control performed by Genomed S.A. DNA integrity was confirmed during library preparation, with no evidence of degradation. Although absorbance-based purity ratios (A260/280 and A260/230) were not recorded, successful PCR amplification of the V3–V4 region and efficient library preparation indicated that the DNA exhibited sufficient purity and lacked inhibitory compounds.

### 2.4. Library Preparation and Sequencing

An amplicon-based 16S rRNA gene analysis of the bacterial population was performed based on the hypervariable V3-V4 region of the 16S rRNA gene. Specific primers 341F and 785R with Illumina adapter overhang sequences were used to amplify the selected region and prepare the sequencing library. The primer sequences were as follows:

341F:5′*TCGTCGGCAGCGTCAGATGTGTATAAGAGACAG*CCTACGGGNGGCWGCAG

785R:5′*GTCTCGTGGGCTCGGAGATGTGTATAAGAGACAG*GACTACHVGGGTATCTAATCC

The italicized parts correspond to the Illumina adapter overhangs, while the bold sequences target the V3-V4 region of the 16S rRNA gene [29]. All steps, including amplification, indexing, and library quantification, followed the Illumina “16S Metagenomic Sequencing Library Preparation” protocol. PCR amplification was performed using Q5 Hot Start High-Fidelity 2X Master Mix (New England Biolabs, Ipswich, MA, USA) under the following conditions: -initial denaturation: 95 °C for 3 min, -25 cycles of: 95 °C for 30 s, 55 °C for 30 s, 72 °C for 30 s, -final extension: 72 °C for 5 min and hold at 4 °C. The resulting amplicons were indexed with the Nextera XT Index Kit (Illumina, San Diego, CA, USA). Library size was assessed using the Agilent Bioanalyzer 2100 with a DNA High Sensitivity chip. Sequencing was performed on the Aviti platform (Element Biosciences, San Diego, CA, USA) in paired-end 2 × 300 nt mode, targeting at least 50,000 read pairs per sample.

### 2.5. Bioinformatics Analysis

Taxonomic classification to the species level was conducted using QIIME 2 software (2024.2) with the SILVA 138 reference database. The DADA2 pipeline was employed to distinguish true biological sequences from sequencing errors and to identify unique amplicon sequence variants (ASVs). For phylogenetic analysis, ASVs were aligned using the MAFFT algorithm, and a phylogenetic tree was constructed with the FastTree method. Further bioinformatics analyses were performed in R, utilizing the phyloseq [30] and vegan [31] packages. Visualization and charting were done with ggplot2 [32], gplots [33], plotly [34] and heatmaply [35] packages. The 16S rRNA amplicon sequencing analysis was performed at Genomed S.A. (Warsaw, Poland). Paired-end reads were processed in QIIME 2 using the dada2 denoise-paired approach. Quality profiles were inspected prior to trimming, and parameters were selected according to Illumina recommendations for V3–V4 amplicons (expected size ~550 bp). The following DADA2 settings were used: trim-left-f: 17, trim-left-r: 21, trunc-len-f: 280, trunc-len-r: 250, max-ee: 2, trunc-q: 2, and chimera-method: consensus. These trimming and filtering thresholds ensured removal of low-quality bases, maintained sufficient paired-end overlap (>50 bp), and allowed accurate inference of amplicon sequence variants (ASVs).

### 2.6. Statistical Analysis

All culture-based methods were performed in triplicate, and results were expressed as logarithmic colony-forming units per gram (log CFU/g). Results reported as “below the detection or quantification limit” (e.g., <1 log CFU/g, according to ISO standards [22,23,24,25,26]) were incorporated into the statistical analysis using a commonly applied substitution approach. Specifically, values below the limit of quantification (LOQ) were replaced by one-half of the LOQ value (LOQ/2). In this study, where the LOQ corresponded to 1 log CFU/g, results reported as “<1 log CFU/g” were therefore entered as 0.5 log CFU/g for statistical purposes. This approach assumes an average microbial load between zero and the detection limit and is widely used in environmental, microbiological, and toxicological analyses, in line with EFSA and FAO/WHO guidance on handling left-censored data [36,37,38]. Because the data included substituted values (<LOQ) and did not meet normality assumptions, a non-parametric Kruskal–Wallis test was used to compare microbial counts among cheese samples (ZJ-473, ZJ-504, ZJ-505, ZJ-513) for each microbial group. When the overall effect was significant (*p* < 0.05), Dunn’s post hoc test with Bonferroni correction was applied to identify pairwise differences. The effect size (ε^2^) was calculated for each Kruskal–Wallis test to quantify the strength of group differences. All analyses were conducted in R version 4.5.1 (R Core Team, 2025) using the packages tidyverse (for data management and visualization) [39], rstatix (for Kruskal–Wallis and effect-size calculations) [40], rcompanion (compact letter display) [41], purr (functional programming tools) [42] and ggbeeswarm (jittered point plots) [42]. For co-occurrence network analysis, pairwise correlations between bacterial taxa were calculated using Spearman’s rank correlation coefficient. Only statistically significant associations with *p* < 0.05 and an absolute correlation coefficient of |r| ≥ 0.6 were retained for network construction.

## 3. Results

### 3.1. Culture-Based Methods

**Figure 1:**
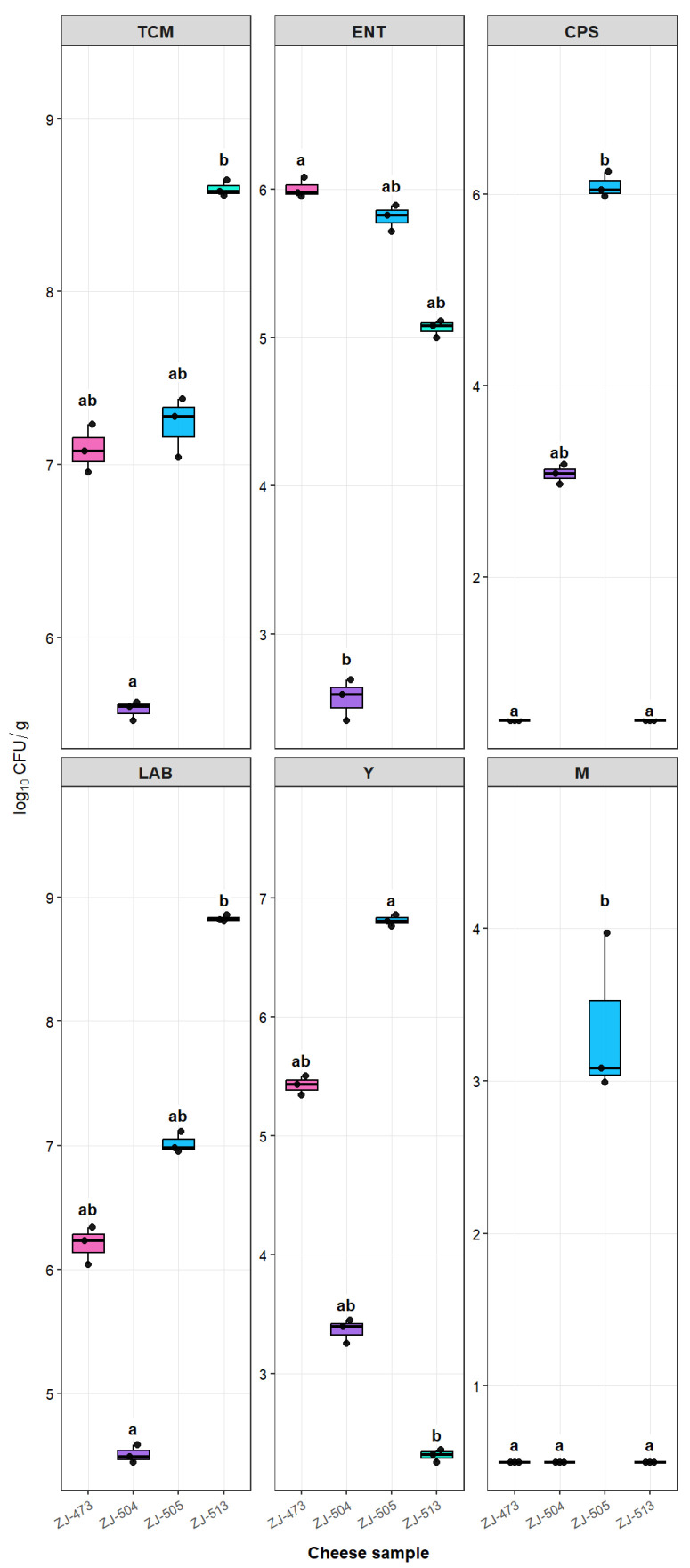
Microbiological counts of six microbial groups in cheese samples. Boxplots show the microbiological counts (log_10_ CFU/g) of six microbial groups: TCM—total count of microorganisms; ENT—*Enterobacteriaceae*; CPS—coagulase-positive staphylococci; LAB—lactic acid bacteria; Y—yeasts; and M—molds, in cheese samples (ZJ-473, ZJ-504, ZJ-505, and ZJ-513). Each value represents the mean of triplicate culture-based determinations. Results below the quantification limit (<1 log CFU/g) were replaced by LOQ/2 (0.5 log CFU/g) for statistical analysis. Different lowercase letters above the boxes indicate significant differences among cheeses within the same microbial group, as determined by the Kruskal–Wallis test followed by Dunn’s post hoc test with Bonferroni correction (*p* < 0.05).

The microbiological counts of the examined cheese samples are summarized in Table 1 and visualized in Figure 1. Overall, significant differences (*p* < 0.05) were observed among cheeses for all analyzed microbial groups, except where indicated. TCM and LAB were the highest in samples ZJ-473, ZJ-505 and ZJ-513, indicating a more advanced fermentation process or higher microbial activity in these products. In contrast, ZJ-504 exhibited the lowest counts for most microbial groups, reflecting comparatively lower microbial load. *Enterobacteriaceae* (ENT) were detected at moderate levels in samples ZJ-473, ZJ-505, and ZJ-513, whereas they remained minimal in ZJ-504. Coagulase-positive staphylococci (CPS) were found only in samples ZJ-504 and ZJ-505, with counts exceeding 3 and 6 log CFU/g, respectively, while remaining below the detection limit in the other cheeses. Yeasts and molds (Y and M) followed similar trends, being markedly more abundant in ZJ-505 and almost absent in ZJ-473, ZJ-504, and ZJ-513.

### 3.2. 16S rRNA Amplicon Sequencing

**Figure 2:**
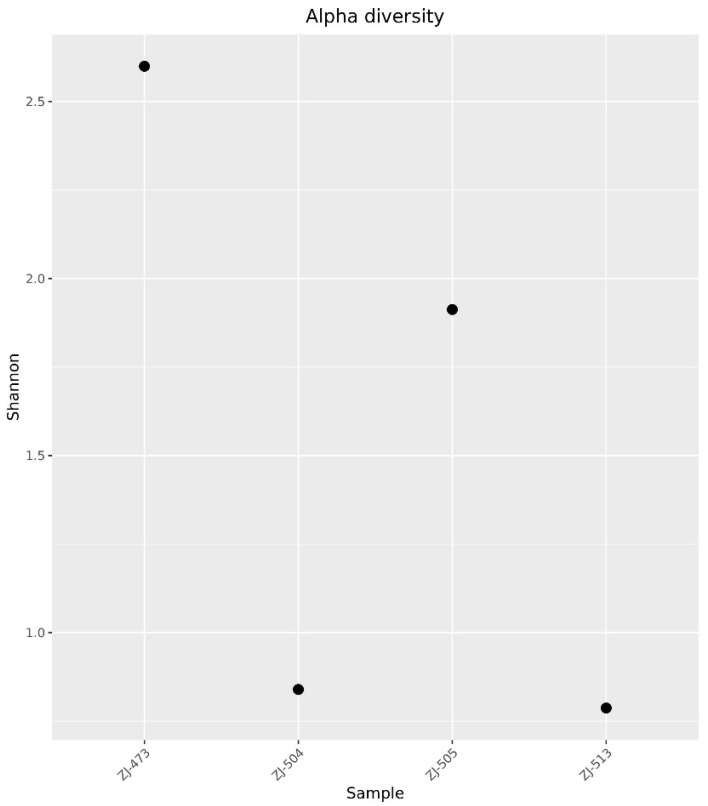
Alpha diversity of bacterial communities based on the Shannon index at the species level. Each point represents the microbial diversity of a cheese sample obtained from one of four dairies (ZJ-473, ZJ-504, ZJ-505, and ZJ-513). Higher Shannon index values indicate greater richness and evenness within the bacterial community, reflecting differences in overall microbial diversity among samples.

Bacterial community richness and evenness were estimated by measuring alpha diversity using the Shannon index. The alpha diversity of microbial communities varied substantially among the cheese samples (Figure 2). The highest diversity was observed in sample ZJ-473 (H’ ≈ 2.6), indicating a more complex and evenly distributed microbial community. Moderate diversity was found in ZJ-505 (H’ ≈ 1.9), whereas samples ZJ-504 and ZJ-513 exhibited low Shannon indices (H’ < 1.0), suggesting a dominance of few taxa and reduced community richness.

**Figure 3:**
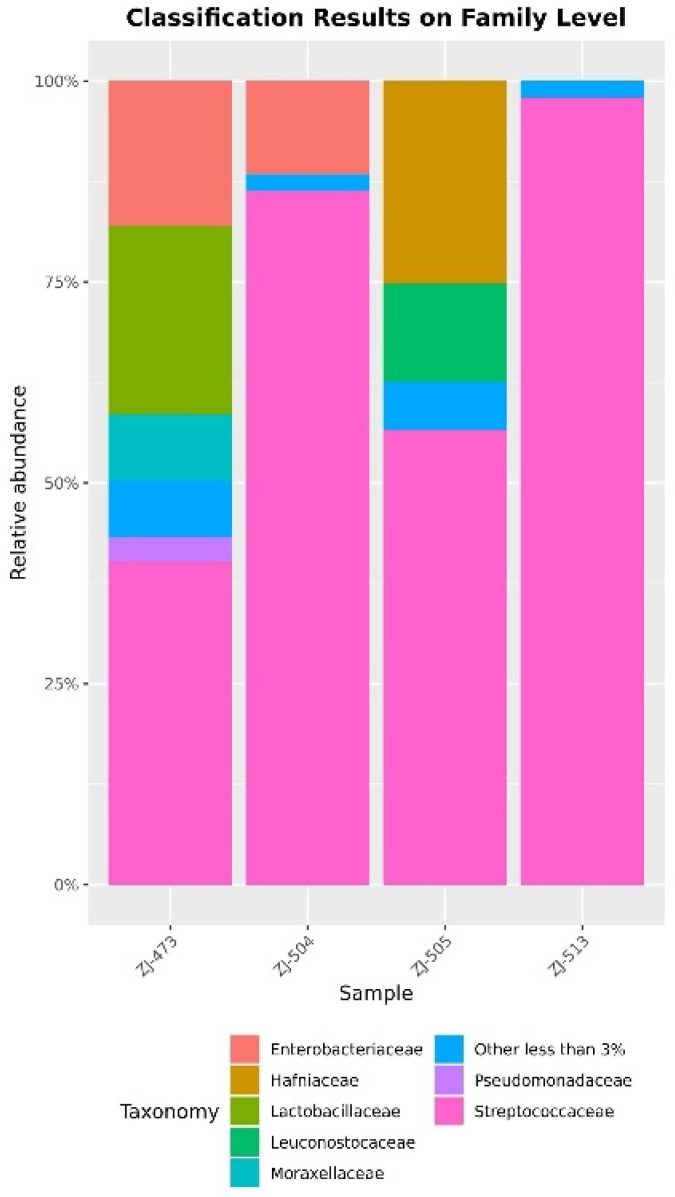
Taxonomic composition of bacterial communities at the family level. Stacked bar charts present the relative abundance of bacterial families identified in cheese samples (ZJ-473, ZJ-504, ZJ-505, and ZJ-513). Each bar represents the proportion of individual bacterial families within the total microbial community of a given sample. Taxa with a relative abundance <1% were merged into the category ‘Other less than 3%’.

**Figure 4:**
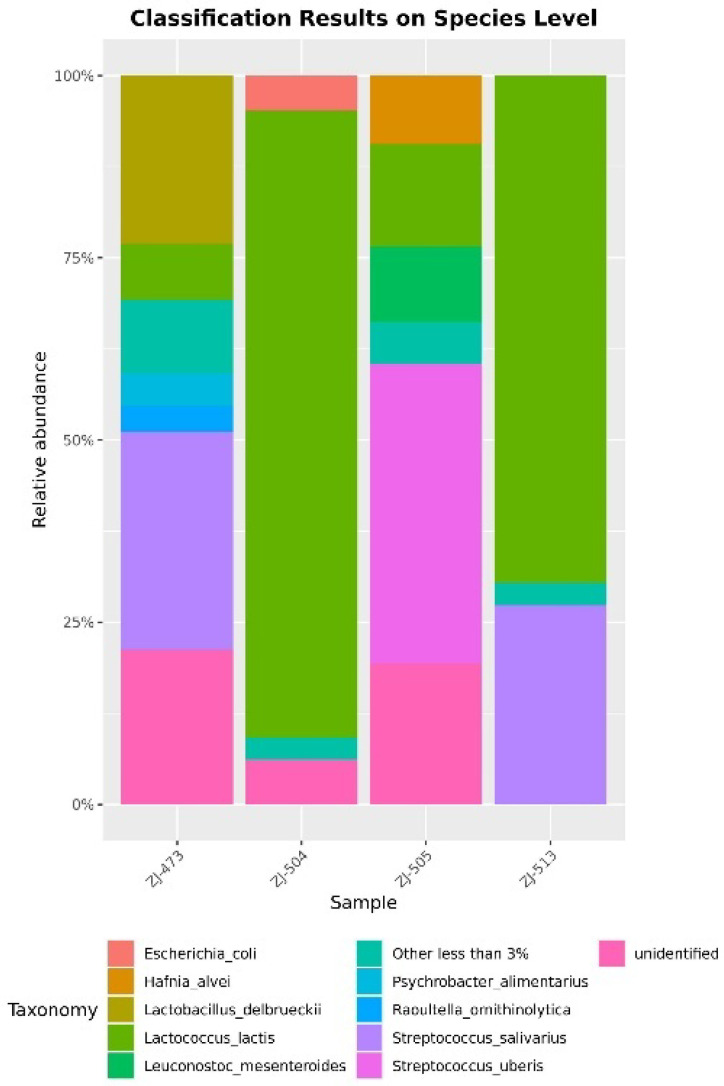
Taxonomic composition of bacterial communities at the species level. Stacked bar charts present the relative abundance of bacterial species identified in cheese samples (ZJ-473, ZJ-504, ZJ-505, and ZJ-513). Each bar represents the proportion of individual bacterial species within the total microbial community of a given sample. Taxa with a relative abundance <1% were merged into the category ‘Other less than 3%’.

The taxonomic composition of the bacterial communities was examined at both the family and species levels (Figure 3 and Figure 4). At the family level, the microbial profiles of the cheese samples were dominated by members of the *Streptococcaceae* family, particularly in samples ZJ-504, ZJ-505, and ZJ-513, where this group accounted for the majority of the relative abundance. *Lactobacillaceae* and *Leuconostocaceae* were also detected in ZJ-473 and ZJ-505, while *Enterobacteriaceae* and *Hafniaceae* appeared as minor but distinct components of the microbiota in certain samples. At the species level, clear differences in microbial structure were observed among the samples. Sample ZJ-473 showed the highest taxonomic diversity, containing several taxa such as *Streptococcus uberis*, *Lactococcus lactis*, and *H. alvei* with comparable abundances. Minor fractions of taxa representing less than 3% of total abundance, as well as sequences that could not be assigned to a specific species, were detected in all samples.

**Figure 5:**
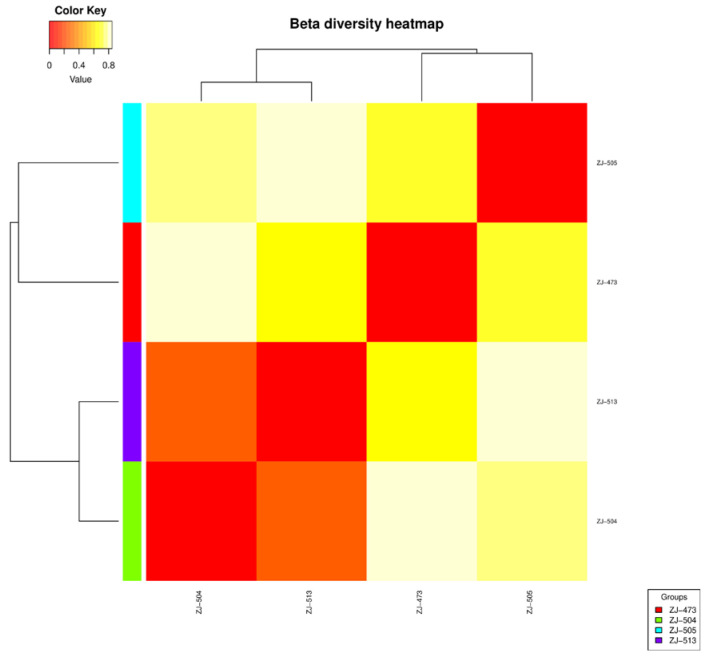
Beta diversity heatmap of the analyzed samples. The heatmap depicts pairwise dissimilarities in microbial community composition based on beta diversity analysis. The color gradient from red to light yellow represents increasing dissimilarity, with red indicating more similar and yellow indicating more distinct communities.

The beta diversity heatmap (Figure 5) illustrates the pairwise dissimilarities among the analyzed samples, providing insight into differences in microbial community composition across treatments. The color gradient from dark red to light yellow corresponds to increasing dissimilarity values, with red indicating more similar community structures (low distance values) and yellow representing greater divergence (high distance values). Clear clustering patterns are observed among the samples. ZJ-504 and ZJ-513 show the highest similarity, as indicated by their close proximity in the dendrogram and the predominance of red tones between them. Sample ZJ-473 exhibits moderate similarity, clustering near ZJ-504 and ZJ-513 and showing intermediate color values. In contrast, ZJ-505 differs markedly from all others, forming a separate branch and showing lighter (yellow) pairwise colors, reflecting its distinct microbial composition. Overall, the heatmap reveals a primary cluster composed of ZJ-504, ZJ-513, and ZJ-473, and one distinct outlier, ZJ-505, highlighting pronounced beta-diversity differences among the analyzed groups.

**Figure 6:**
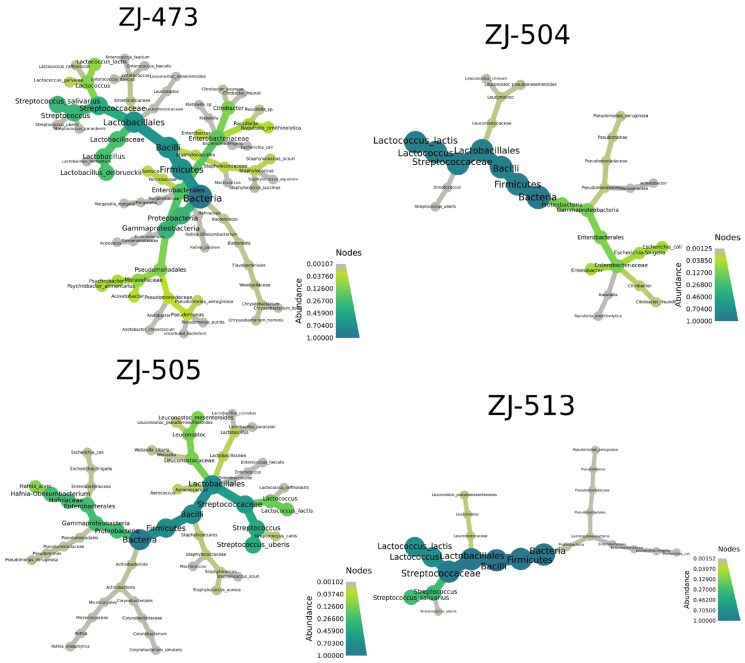
Co-occurrence network diagrams of bacterial taxa identified in cheese samples. Network representations show bacterial taxonomic relationships within cheese samples ZJ-473, ZJ-504, ZJ-505, and ZJ-513. Node size corresponds to relative abundance, and node color indicates abundance intensity according to the color scale. Edges represent co-occurrence links among taxa, highlighting associations within each sample’s microbial community structure. The networks illustrate the taxonomic complexity and connectivity patterns characteristic of bacterial communities at the species level in the analyzed cheeses.

The co-occurrence network analysis revealed distinct structural patterns and levels of complexity among the bacterial communities in the analyzed cheese samples (Figure 6). In all networks, nodes represented bacterial taxa and edges indicated co-occurrence relationships, with node size and color corresponding to relative abundance.

**Figure 7:**
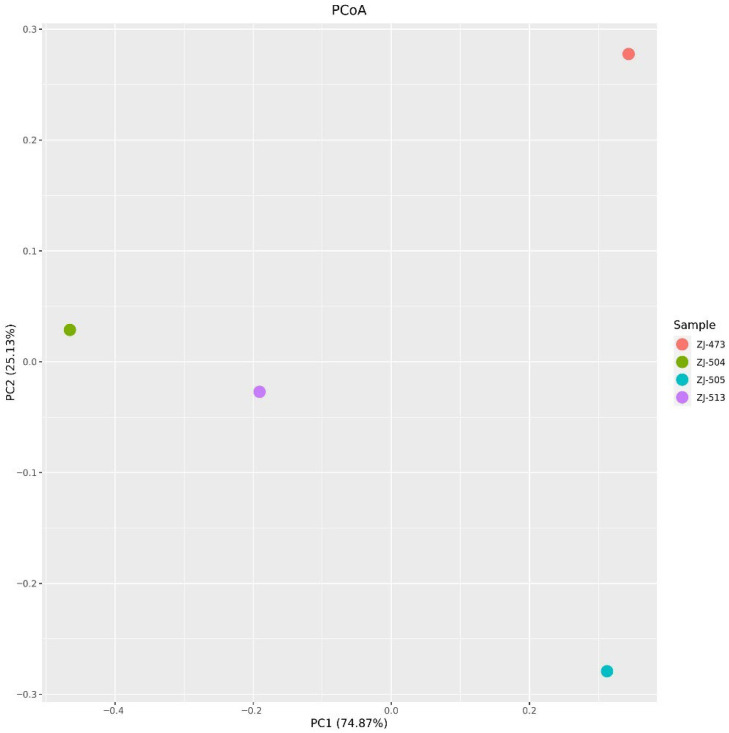
Principal coordinates analysis (PCoA) of bacterial community composition in cheese samples. The PCoA plot visualizes differences in bacterial community structure among cheese samples (ZJ-473, ZJ-504, ZJ-505, and ZJ-513) based on beta diversity metrics. Each point represents an individual sample, and distances between points correspond to compositional dissimilarities. The first two principal coordinates (PC1 and PC2) explain 74.87% and 25.13% of the total variance, respectively, indicating clear separation among samples and distinct microbial community profiles.

The principal coordinates analysis (PCoA) based on beta diversity metrics revealed clear separation among the bacterial communities of the analyzed cheese samples (Figure 7). The first two coordinates explained 74.87% and 25.13% of the total variance, respectively, accounting for nearly all variation observed in the dataset. Distinct clustering patterns were observed for each sample, indicating differences in microbial community composition. Sample ZJ-473 was positioned apart from the others along the positive axis of PC1, while ZJ-505 was separated along the negative axis of PC2.

**Figure 8:**
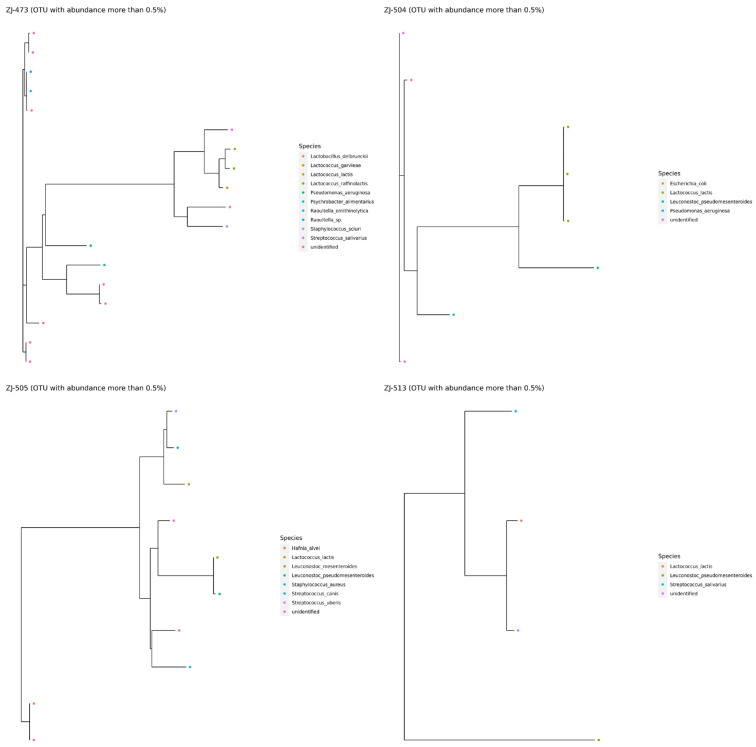
Phylogenetic relationships of bacterial ASVs with relative abundance above 0.5% in cheese samples. Phylogenetic trees show the distribution and relationships of dominant bacterial ASVs identified in cheese samples ZJ-473, ZJ-504, ZJ-505, and ZJ-513. Each colored dot represents a distinct bacterial species, with colors corresponding to taxa listed in the legend.

Phylogenetic trees illustrating amplicon sequence variants (ASVs) with relative abundance above 0.5% revealed clear differences in bacterial community composition among the analyzed cheese samples (Figure 8). Sample ZJ-473 exhibited the most complex phylogenetic structure, with a diverse range of species belonging primarily to the genera *Lactococcus*, *Lactobacillus*, *Streptococcus*, *Raoultella*, and *Pseudomonas*. The presence of multiple *Lactococcus* species, including *L. lactis*, *L. garvieae*, and *L. raffinolactis*, alongside *Lactobacillus delbrueckii* and *Streptococcus salivarius*, indicated a well-developed community of LAB complemented by occasional environmental and opportunistic taxa. In contrast, sample ZJ-504 showed a simpler community, dominated by *Lactococcus lactis* and *Leuconostoc pseudomesenteroides*, with only a few additional species such as *Pseudomonas aeruginosa* and *Escherichia coli* present. The phylogenetic tree for ZJ-505 revealed intermediate diversity, including *Lactococcus lactis*, *Leuconostoc* spp., *H. alvei*, *Staphylococcus aureus*, and several *Streptococcus* species. Sample ZJ-513 displayed the lowest diversity, comprising primarily *Lactococcus lactis*, *L. pseudomesenteroides*, and *S. salivarius*.

## 4. Discussion

The complementary use of conventional culture-based enumeration and 16S rRNA amplicon sequencing provided a holistic assessment of microbial communities in artisanal cheese samples (ZJ-473, ZJ-504, ZJ-505, and ZJ-513). Culture-based methods remain essential for quantifying viable microorganisms and verifying compliance with food safety criteria, whereas 16S rRNA amplicon-based profiling reveals the total bacterial diversity, including unculturable or low-abundance taxa [22,23,24,25,26,43]. Similar integrative strategies have been applied to evaluate the microbiota of traditional European cheeses, enhancing understanding of microbial ecology and safety [10,11,12]. Recent studies confirm that combining culture and sequencing data strengthens microbial risk assessments and facilitates the identification of both beneficial and potentially hazardous taxa [43,44]. Although our sampling was restricted to a single voivodeship, the Łódź region is widely regarded as representative of Central Poland in terms of geography, farm structure, and artisanal cheese production; therefore, the observed patterns of microbial diversity are likely to reflect broader trends in raw-milk “twarog” cheeses from this part of the country.

Alpha-diversity analyses confirmed clear differences in microbial complexity among cheeses, with ZJ-473 showing the richest and most evenly distributed microbiota. In contrast, ZJ-504 and ZJ-513 were strongly dominated by *Lactococcus lactis*, reflecting reduced evenness and lower overall diversity, while ZJ-505 displayed an intermediate community structure consisting of *L. lactis*, *S. uberis* and *L. mesenteroides*. These findings further illustrate notable variability in microbiological quality and microbial community composition among the cheeses, likely reflecting differences in production conditions and hygiene management practices. The beta-diversity heatmap further supported these patterns, revealing one main cluster (ZJ-504, ZJ-513, ZJ-473) and a distinct outlier (ZJ-505), indicating clear between-sample compositional differences. The PCoA ordination supported these observations, with ZJ-504 and ZJ-513 clustering closer together, indicating partial similarity in their bacterial communities, whereas the overall spatial distribution further illustrated substantial variability in community structure across cheeses originating from different dairies. Co-occurrence network analysis reinforced these patterns, revealing that ZJ-473 and ZJ-505 exhibited the most complex clusters dominated by *Lactococcus*, *Streptococcus* and *Lactobacillus*, alongside *Proteobacteria* such as *Enterobacteriaceae* and Pseudomonadaceae. In contrast, networks in ZJ-504 and ZJ-513 showed fewer nodes and weaker connectivity, reflecting simplified microbial communities dominated by LAB with sporadic environmental taxa. Overall, these network structures, ordination patterns and phylogenetic signals confirm substantial variation in bacterial community richness and taxonomic structure among the cheeses, with ZJ-473 and ZJ-505 exhibiting the most diverse microbiota, whereas ZJ-504 and ZJ-513 displayed more simplified LAB-dominated assemblages.

Certain LAB naturally occurring in raw milk produce bacteriocins, antimicrobial peptides synthesized by the ribosome, which suppress the growth of pathogenic microbes. Based on their structural and functional properties, bacteriocins are categorized into three main classes. Antibiotics such as nisin, produced by *Lactococcus lactis*, have been extensively studied for their activity against foodborne pathogens like *Staphylococcus aureus* and *Listeria monocytogenes*. Likewise, *Enterococcus* species isolated from dairy environments can produce bacteriocins with strong anti-*Listeria* effects [8,13]. In addition to bacteriocins, LAB synthesize a range of antimicrobial compounds, including organic acids and hydrogen peroxide, which contribute to the inhibition of pathogenic microorganisms. *Lactococcus garvieae*, frequently isolated from raw milk cheeses, contributes to microbial stability by producing bacteriocins and hydrogen peroxide, which inhibit pathogens such as *S. aureus*. Despite its association with infections in animals and humans, its presence in cheese has shown beneficial effects without compromising sensory quality. *Enterococcus* spp., notably *E. durans*, play a functional role during cheese ripening by producing antimicrobial compounds effective against *Bacillus cereus*, *S. aureus*, *Escherichia coli*, *L. monocytogenes*, and *Enterococcus faecalis*, while also enhancing flavor development. *Hafnia* spp., such as *H. alvei*, are involved in flavor formation through sulfur compound production and act as adjunct ripening cultures. They support microbial balance by suppressing *E. faecalis* and *E. coli* O26:H11, highlighting their potential as functional cultures in raw milk cheese production [8,13,14,15,16]. Recent studies have provided updated insights into the microbial diversity, ecological interactions and safety-related characteristics of traditional raw-milk cheeses, broadening current understanding of how cheese-associated microbiota develop and influence product quality and safety [17,18,19,20,21].

Microbiological counts showed distinct quantitative differences among samples. LAB dominated all cheeses, confirming their essential role in fermentation and microbial stability [13,14]. The highest LAB levels observed in ZJ-473 and ZJ-505 indicated more active fermentation, while ZJ-504 and ZJ-513 displayed reduced bacterial activity, potentially due to milk microbiota. The detection of *Enterobacteriaceae* and coagulase-positive staphylococci in certain samples suggested minor hygiene deviations during processing, as also reported for small-scale Polish and Mediterranean cheeses [4,5,15]. The statistical handling of left-censored data (values below LOQ) followed EFSA guidance and best practices for quantitative microbial risk assessment [36,37,38]. These procedures, together with ISO-standardized enumeration methods, ensure the comparability and regulatory validity of the results.

From a food safety perspective, the microbial profiles observed in this study highlight both protective and potentially hazardous elements inherent to raw milk cheese production. The absence of *Salmonella* spp. and *Listeria monocytogenes* in all samples suggests that fermentation-driven acidification and LAB-mediated competitive exclusion were effective hurdles [4,5,8]. However, the detection of *Enterobacteriaceae* and particularly the high counts of coagulase-positive staphylococci in one sample (exceeding 5 log) indicate hygiene deviations associated with milking or early processing stages. According to Regulation (EC) No 2073/2005 [45], levels above 5 log CFU/g require additional testing for staphylococcal enterotoxins as part of process hygiene verification. Although toxin detection was outside the scope of this study, the result underscores the need for strengthened hygiene control at critical steps, a risk consistently documented in artisanal dairy systems [4,5,10,11,12]. It should be noted that enumeration of coagulase-positive staphylococci does not distinguish between enterotoxigenic and non-enterotoxigenic *S. aureus*, as ISO 6888-2 detects coagulase activity rather than toxin production or pathogenicity markers [8,45]. In accordance with Commission Regulation (EC) No 2073/2005, CPS counts are therefore interpreted as a process hygiene criterion, and levels above 5 log CFU/g require verification through enterotoxin testing rather than indicating pathogenic strains per se [45]. Based on EFSA One Health assessments [6,7] and artisanal dairy hygiene guidance [9], the key hazards in raw milk cheese production arise from environmental contamination, insufficient sanitation, and manual handling. Reinforcing raw milk quality monitoring (TBC, SCC), ensuring strict clean–dirty separation, sanitizing equipment thoroughly, and performing periodic microbiological checks of finished cheeses are therefore essential. Maintaining a dominant LAB community naturally occurring or supported by autochthonous cultures may further limit growth of opportunistic contaminants [11,13,14,15,16]. Overall, our findings show that artisanal raw milk cheeses can be produced safely, provided that hygiene management and ongoing risk assessment are applied consistently.

Amplicon sequencing revealed substantial variation in community diversity and structure among samples. Alpha diversity (Shannon index) was highest in ZJ-473 and ZJ-505, whereas ZJ-504 and ZJ-513 exhibited simplified communities. Beta diversity (PCoA) clearly separated the samples, indicating dairy-specific microbial signatures (Figure 7). These findings align with previous work on regional raw-milk cheeses such as Serpa PDO and Colonial-type cheese, where local production practices strongly shape microbial assemblages. In Serpa PDO cheeses, dominant microbiota include *Lactobacillus*, *Leuconostoc* and *Enterococcus* species, reflecting sheep-milk fermentation under region-specific ripening conditions, whereas Brazilian Colonial-type cheeses typically contain higher proportions of *Streptococcus* and *Lactococcus*, shaped by small-farm practices and distinct environmental microbiota [10,12]. Recent large-scale studies confirm that metagenomic diversity is influenced by milk source, ripening time, and environmental microbiota [43,44,46,47]. The pronounced compositional differences in our samples are consistent with observations from Polish Koryciński cheese [4], where raw-milk microbiota and handling hygiene were major determinants of bacterial diversity.

The taxonomic profiles based on 16S rRNA amplicon sequencing were dominated by *Firmicutes* and *Proteobacteria*, with *Lactococcus lactis*, *Leuconostoc pseudomesenteroides*, *L. delbrueckii*, and *S. salivarius* being the most prevalent species typical of raw-milk cheeses [8,13]. *Staphylococcus aureus*, *H. alvei*, and *Pseudomonas aeruginosa* were sporadically detected, reflecting potential environmental contamination or post-ripening exposure [5,15]. Phylogenetic analysis highlighted that ZJ-473 possessed the most diverse bacterial structure, while ZJ-504 and ZJ-513 were dominated by a limited set of LAB species. Such variation mirrors reports from raw-milk cheeses across Europe and South America, where microbial diversity depends on production scale and ecological stability [10,16]. The coexistence of beneficial and opportunistic bacteria in raw-milk cheeses has also been observed in recent EU and Polish investigations [4,5,10], supporting the need for regular microbiological monitoring.

Network analysis revealed more complex and connected microbial communities in ZJ-473 and ZJ-505 than in ZJ-504 and ZJ-513 (Figure 6). These denser interaction webs suggest greater ecological stability and metabolic cross-feeding among LAB species. LAB such as *Lactococcus lactis* and *L. delbrueckii* contribute to ecosystem balance by producing organic acids, hydrogen peroxide, and bacteriocins that inhibit spoilage organisms [11,14]. Similar associations were described in recent ecological network studies of traditional dairy fermentations [44,48,49], which linked microbial connectivity to product consistency and functional resilience. Our findings align with this concept, showing that cheeses with richer and more interactive microbiota (ZJ-473, ZJ-505) are likely to possess stronger self-stabilizing ecosystems and more complex ripening dynamics.

Combining culture-based and sequencing results provides a comprehensive understanding of microbial safety in raw-milk cheeses. The dominance of LAB observed in both datasets indicates a protective microbiota capable of acidification and competitive exclusion of pathogens [11,13,47]. Nevertheless, the detection of *Staphylococcus aureus* and *Enterobacteriaceae* in certain samples, even at low abundance, reinforces the need for hygiene management and continuous risk assessment [4,5]. The European Commission’s biosafety guidance for artisanal cheeses [9] and the most recent EFSA–ECDC One Health Zoonoses reports [6,7] highlight *Listeria monocytogenes*, *Salmonella* spp., and *Staphylococcus aureus* as the principal microbiological hazards in unpasteurized dairy products. Integrating 16S rRNA amplicon-based microbial profiling with classical microbiological testing, as demonstrated in this study, supports early detection of such risks and complements standard HACCP procedures [44].

## 5. Conclusions

Overall, the combined culture-based and amplicon-based sequencing approach revealed substantial variability in microbial diversity, composition, and ecological complexity among the analyzed cheese samples. ZJ-473 and ZJ-505 displayed highly diverse and interconnected microbial ecosystems, while ZJ-504 and ZJ-513 contained simpler, LAB-dominated communities. These differences likely stem from variations in raw-milk microbiota, hygienic practices, and ripening conditions. Future research should integrate multi-omics approaches linking metagenomics, metabolomics, and metatranscriptomics to elucidate how microbial structure influences sensory properties, technological performance, and safety. Expanding such integrated analyses will enhance understanding of microbial ecology in artisanal cheeses and contribute to improved process control and consumer protection [43,47,49]. The results of the research presented in this article contributed to the isolation of autochthonous LAB from the analyzed cheeses, which were subsequently used in the development of starter cultures for the production of cheeses made from raw milk.

## Figures and Tables

**Table 1:** Microbiological counts and detection of pathogenic bacteria in cheese samples. Table 1 presents the microbiological counts (log_10_ CFU/g) determined in cheese samples (ZJ-473, ZJ-504, ZJ-505, and ZJ-513) for each microbial group: TCM—total count of microorganisms; ENT—*Enterobacteriaceae*; CPS—coagulase-positive staphylococci; LAB—lactic acid bacteria; Y—yeasts; and M—molds. The presence of pathogenic bacteria was also assessed for SALM—*Salmonella* spp. and LIST—*L. monocytogenes*; nd—not detected. Values are expressed as medians with interquartile ranges (Q1–Q3), based on triplicate culture-based determinations. Within each microbial group, different lowercase letters indicate statistically significant differences among cheese samples, as determined by the Kruskal–Wallis test followed by Dunn’s post hoc test with Bonferroni correction (*p* < 0.05). The use of medians and non-parametric tests reflects the non-normal distribution of microbial counts and the occurrence of values below the detection limit.

Analysis	Cheese Symbol			
	ZJ-473	ZJ-504	ZJ-505	ZJ-513
TCM [log CFU g^−1^]	7.08 [7.02–7.15] ^ab^	5.6 [5.56–5.61] ^a^	7.28 [7.16–7.33] ^ab^	8.58 [8.57–8.61] ^b^
ENT [log CFU g^−1^]	5.98 [5.97–6.03] ^a^	2.59 [2.50–2.64] ^b^	5.83 [5.77–5.86] ^ab^	5.08 [5.04–5.10] ^ab^
CPS [log CFU g^−1^]	0.50 [0.50–0.50] ^a^	3.08 [3.03–3.13] ^ab^	6.04 [6.01–6.14] ^b^	0.50 [0.50–0.50] ^a^
LAB [log CFU g^−1^]	6.23 [6.14–6.29] ^ab^	4.49 [4.47–4.54] ^a^	6.98 [6.97–7.05] ^ab^	8.82 [8.81–8.84] ^b^
Y [log CFU g^−1^]	5.43 [5.39–5.47] ^ab^	3.40 [3.33–3.42] ^ab^	6.81 [6.78–6.83] ^a^	2.32 [2.29–2.34] ^b^
M [log CFU g^−1^]	0.50 [0.50–0.50] ^a^	0.50 [0.50–0.50] ^a^	3.08 [3.03–3.52] ^b^	0.50 [0.50–0.50] ^a^
SALM	nd	nd	nd	nd
LIST	nd	nd	nd	nd

## Data Availability

The original contributions presented in this study are included in the article. Further inquiries can be directed to the corresponding author.
